# The impacts of national centralized drug procurement policy on drug utilization of medical institutions: an empirical study in a county-level hospital in China

**DOI:** 10.1186/s12913-024-10964-7

**Published:** 2024-04-24

**Authors:** Haoye Li, Fanyu Lin, Rui Wang, Chenxuan Zhu, Keyao Cao, Yu Chen, Gang Fang, Jiaming Li, Jinxi Ding, Wei Li

**Affiliations:** 1 School of International Pharmaceutical Business, China Pharmaceutical University, Nanjing, China; 2 Pharmaceutical Market Access Policy Research Center, China Pharmaceutical University, Nanjing, China

**Keywords:** County-level hospital, Drug utilization, Influencing factors, National centralized drug procurement policy

## Abstract

**Purpose:**

Under the background of the regular implementation of the National Centralized Drug Procurement (NCDP) policy, this study aimed to assess the impacts of the NCDP policy on drug utilization of county-level medical institutions, and probe into the influencing factors of the changes in drug utilization.

**Method:**

A pre-post study was applied using inpatient data from a county-level medical institution in Nanjing. Drug utilization behavior of medical institutions of 88 most commonly used policy-related drugs (by generic name, including bid-winning and bid-non-winning brands) was analyzed, and the substitution of bid-winning brands for brand-name drugs after policy intervention was evaluated.

**Results:**

After policy intervention, 43.18% of policy-related drugs realized the substitution of bid-winning brands for bid-non-winning brands (6.82% of complete substitution, 36.36% of partial substitution). Meanwhile, 40.90% of policy-related drugs failed to realize brand substitution. Multiple factors affected brand substitution, including: (1) Policy effect: brand substitution was more obvious after the intervention of the first and third round of NCDP. (2) Drug market competition: the greater the price reduction of bid-non-winning brands, the more the drugs for the same indication, the more likely that medical institutions keep using the same brands as they did before policy intervention. (3) Previous drug utilization of medical institutions: brand substitution was more obvious in drugs with large number of prescriptions and weak preference for brand-name drugs.

**Conclusion:**

The NCDP policy promoted the substitution of bid-winning brands for bid-non-winning brands. However, the NCDP policy remained to be further implemented in county-level medical institutions. Policy implememtation efforts, drug market competition and drug utilization of medical institutions would affect the implementation of the NCDP policy.

## Introduction

National Centralized Drug Procurement (NCDP) policy is one of the supplemental drug procurement policies in China implemented since December 2018. Since 2009, China has initiated healthcare reform, and implemented a province-based, government-led procurement pattern, whereby enterprises negotiate with medical institutions after listing on the provincial-level platform and medical institutions purchase on-demand. In December 2018, the State Council released the *Pilot Program for National Centralized Drug Procurement and Utilization*, which introduced NCDP policy for the first time, supplementing the existing drug procurement pattern in China. By October 2023, there have been eight rounds of centralized procurement, with an average of 41 policy-related drugs per round and an average price reduction of more than 50% [1].

NCDP policy is a government-led procurement pattern, which is widely practiced around the world. For example, the UK has a universal health insurance system, with the NHS (National Health Service) leading the centralized procurement of off-patent drugs and generic drugs in public hospitals [2, 3]. In Hong Kong China, drug is centrally procured by the Hospital Authority (HA) of SAR (Special Administrative Region) in conjunction with all the public healthcare institutions [4].

Led and organized by the National Healthcare Security Administration (NHSA), the NCDP policy is implemented through a comprehensive service platform.^1^ NHSA selects drugs with sufficient market competition and large market scale, negotiates prices with enterprises (no distinction between brand-name drug and generic drug) based on their quoted prices, supply capacity, market recognition and other comprehensive conditions. The bid-winning enterprise reduces its price drastically, and in order to guarantee its benefits, NHSA promises 50%—70% of the total annual drug utilization volume of all public medical institutions in the alliance regions (different proportions are set according to the characteristics of drugs).

The NCDP policy, as a supplementary procurement policy, only covers the most commonly used drugs in the clinical setting, and for the first time, mandates public medical institutions to equip a certain volume of the procured brands within a procurement cycle. In addition, the NCDP policy establishes rewards and penalties for medical institutions' drug utilization. For example, additional incentives may be provided based on the completion status of medical institutions, while those who fail to equip the assigned procurement volume may be criticized and reprimanded.

After policy intervention, bid-winning enterprises exhibit two characteristics. On the one hand, the prices of bid-winning brands significantly decrease. Taking Flurbiprofen Ester Injection as an example, the winning enterprise, Wuhan Da'an Pharmaceutical Co., Ltd., witnessed a 64.46% reduction in DDDc (61.77 vs. 21.95, *P* < 0.000), which was significantly lower than bid-non-winning enterprises (21.95 vs. 62.25,^2^*P* < 0.000). Therefore, it is evident that bid-winning enterprises would enjoy a significant pricing advantage.

On the other hand, bid-winning enterprises occupy 50%-70% market share of policy-related drugs in the next procurement cycle. Meanwhile, policy-related drugs also have direct access to medical institutions, which means that after policy intervention, bid-winning enterprises would dominate the market compared to bid-non-winning enterprises.

However, in order to ensure the autonomy of medical institutions in drug procurement and to cope with supply risks, bid-non-winning enterprises would still retain a certain share of the market. Medical institutions can procure drugs from bid-non-winning enterprises under the premise of completing the assigned procurement volume of the bid-winning brands.

Therefore, as a special drug procurement policy, NCDP policy has an impact on the changes of drug utilization of medical institutions in the following three aspects. Firstly, the NCDP policy promoted the substitution of generic drugs for brand-name drugs. Most doctors and pharmacists in China support such substitution based on professional judgment [5]. And there was a significant increase of substitution of generic drugs for brand-name drugs [6, 7]. Secondly, the NCDP policy promoted the substitution of bid-winning brands for bid-non-winning brands. The utilization rate of bid-winning brands in medical institutions increased significantly [8], thus reducing the average cost of medication [9]. Thirdly, the NCDP policy increased the utilization volume of bid-winning brands. For example, NCDP policy has improved the utilization volume of antibiotics [10–12] and drugs for chronic diseases [13, 14].

Current research on drug utilization is relatively sufficient. However, research on NCDP policy is still immature due to limited dimension and depth. Firstly, most studies focus on a certain round of NCDP. Secondly, current research on changes of drug utilization of medical institutions using real-world medication data is insufficient. Most studies use purchasing data which cannot reflect the actual using situation. Thirdly, systematic analysis of the causes of changes in drug utilization brought by NCDP policy is lacking.

Therefore, this research focuses on county-level medical institutions that account for 47.3% of all medical institutions in China. By using inpatient drug utilization data to avoid the impact of the COVID-19, the research cycle consists of six months before the intervention and six months after the intervention to avoid the influence of two rounds of policy-related drugs in the same therapeutic field. This research aims to discover changes in physicians’ drug utilization behavior after the NCDP policy implementation by using one county-level medical institutions inpatient drug utilization data, and explores the factors that affect changes in drug utilization.

## Data and methods

### Data

#### Target varieties

As of carrying out this study, eight rounds of NCDP have been implemented in China. Considering that the sixth round of NCDP only included insulin which featured complex drug substitution, and the seventh and eighth rounds were implemented in Jiangsu in November 2022, August 2023 with a short period, respectively this study only involved drugs of the first to fifth round.

During the defined research cycle, the target medical institution used 103 policy-related drugs, accounting for 47.48% of policy-related drugs involved in the five rounds of NCDP, which was highly representative. However, of the 103 policy-related drugs, 15 had less than 20 prescription records. Such small data volume may result in extreme values, therefore, they were excluded from analysis. Thus, 88 drugs were included, and their brand substitution was analyzed.

#### Research cycle

This research used inpatient data of a county-level medical institution in Nanjing from 1st January 2019 to 31st December 2021. The interval between two rounds of NCDP is about 6 months. Besides, therapeutic areas of policy-related varieties between rounds may be overlapped. Therefore, the research cycle of this study is 12 months for each round, consisting of 6 months before and 6 months after policy implementation, so that interruption between rounds would be avoided (Table 1 Inpatient drug utilization information).

**Table 1:** Inpatient drug utilization information

Round of NCDP	Time of Implementation	Number of Varieties	Research Cycle
1st	23rd December 2019	25	23rd June 2019-23rd June 2020
2nd	27th April 2020	32	27th October 2019-27th October 2020
3rd	1st November 2020	55	1st May 2020-1st May 2021
4th	27th April 2021	45	27th October 2020-27th October 2021
5th	1st November 2021	62	1st May 2021-31st December 2021^a^

^a^The last round is only included one month after implementation due to data limitations

#### Target stata

As this study was launched amid the period of Covid-19, outpatient service in some medical institutions was closed^3^ ^4^, while inpatient service was impacted to a less degree. Therefore, outpatient data was not included in order to maintain the integrity of data. Besides, there is possibility that outpatient patients choose not to purchase drugs in hospital pharmacy, substituting bid-winning drugs for brand-name drugs. Therefore, only inpatient data was used for analysis so that the result could be ensured to reflect the real-world situation.

After desensitizing the patients' personal information and deleting incomplete and abnormal records (volume or amount ≤ 0), a total number of 2,190,677 medication records of 76,284 patients were preserved, including 167,116 records of policy-related drugs (Table 2 Inpatient drug utilization information).

**Table 2:** Inpatient drug utilization information

Data Type	Information Details
Basic information	Item name, item code, dosage form, brand, specification, license number
Drug utilization information	Unit price, drug utilization volume, drug utilization expenses

### Statistical analysis

#### Index

This study focused on the change in price and volume of policy-related drugs after policy intervention.

Drug price was evaluated by Defined Daily Dose Cost (DDDc). DDDc takes DDD as the unit of measurement to reflect the average daily medication cost. The larger the DDDc, the higher the price.


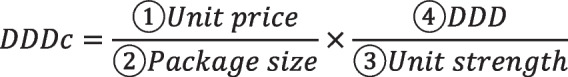

- ①Unit price: sales price of the target drug per package size.
- ②Package size: the minimum quantity of measurement units included in the package unit.
- ③Unit strength: the content of active ingredients in the minimum unit of measurement of the target drug
- ④DDD: Defined Daily Dose, that is, the average daily maintenance dose for adults, determined according to the Guidelines for ATC Classification and DDD Assignment 2021 issued by WHO and the package insert.

Take Acarbose (the second round) for example. Its DDDc of 2.46 is calculated based on the unit price of 36.9 CNY/box, the package size of 30 tablets/box, the unit strength of 0.25ug, and the DDD of 0.5ug.

Drug volume was evaluated by Defined Daily Dose (DDDs). DDDs takes DDD as the unit of measurement to reflect days of application. The larger the DDDs, the larger the volume.


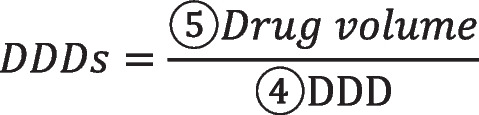

- ⑤Drug volume: the total volume of the target drug by patient in a certain visit.

Take Acarbose (the second round) for example. The DDDs of 30 is calculated by the DDD of 0.5ug and the drug volume of 0.25ug*30 tablets/box*2 boxes.

#### Analytical method

For one thing, the data of this study is not linearly distributed, and it is difficult to choose the control group because NCDP is a nationwide policy. So ITS or DID analysis is not suitable. Eventually, through pre-post study, interrupted by the time of NCDP implementation, this study applied the descriptive statistics to analyze the change in target indexes, and applied rank-sum test or unpaired t test for statistics test.

For another, after grouping the results through descriptive statistics, this study applied rank-Sum test of categorical variables and one-way Anova of continuous variables to launch the between-group test of influencing factors (see Influencing factors of drug utilization change section) so that whether the difference between situations was large could be investigated.

This study used Microsoft Excel 2019 to establish database and used the statistical data analysis software Stata 16.0 to complete the analysis. *p* < 0.05 was considered statistically significant.

## Results

Based on the medication data of 88 policy-related drugs commonly used in medical institutions, this study analyzed the substitution of bid-winning brands for bid-non-winning brands.

### Drug utilization analysis

Through detailed analysis of 167,116 medication data of 88 commonly-used policy-related drugs,^5^ this study summarized the patterns of brand substitution after policy intervention: 43.18% varieties have achieved brand substitution, including high-intensity substitution (complete substitution) and middle-intensity substitution (partial substitution); 40.90% varieties have not achieved brand substitution; 15.91% have achieved alternation of varieties (Table 3 Summary of the substitution of policy-related drugs).

**Table 3:** Summary of the substitution of policy-related drugs

No.	Brand Substitution	Intensity	Situation	Explanation	Proportion	
1	Yes	High	Complete substitution	Bid-non-winning brands were only or partially used before policy intervention, while only bid-winning brands were used after policy intervention	6.82%	43.18%
2		Middle	Partial substitution	Utilization volume of bid-winning brands increased after policy intervention, progressively substituting for bid-non-winning brands	36.36%	
3	No		No substitution	①Bid-winning brands were used both before and after policy intervention, brand selection did not change while dddc and ddds changed; ②DDDc of bid-non-winning brands decreased after policy intervention, medical institutions did not raise the proportion of bid-winning brands	40.90%	40.90%
4	Alternation of varieties		Policy-related drugs came into use after policy intervention	Policy-related drugs were not used before policy intervention and came into use after policy intervention	9.09%	15.91%
5			Policy-related drugs no longer used after policy intervention	Policy-related drugs were no longer used after policy intervention	6.82%	

#### Situation 1: brand substitution

##### High-intensity substitution

High-intensity substitution (complete substitution) referred to the partial or complete utilization of bid-non-winning brands before policy intervention, and complete utilization of bid-winning brands after policy intervention. 6 (6/88, 6.81%) policy-related drugs realized complete substitution (Table 4 Drugs of complete substitution).

**Table 4:** Drugs of complete substitution

No.	Round	Generic Name	Brand	DDDc			DDDs		
				**Before**	**After**	**Rate of Change**	**Before**	**After**	**Rate of Change**
1	First	Tenofovir disoproxil fumarate tablets	Bid-winning		0.47	-97.15%***		45.00	3.25%***
			Bid-non-winning	16.33			43.58		
2	First	Montelukast sodium tablets	Bid-winning		3.79	-32.27%***		11.19	13.22%
			Bid-non-winning	5.60			9.88		
3	Third	Montelukast sodium oral granules	Bid-winning		1.60	-76.45%***		13.17	-2.41%
			Bid-non-winning	6.78			13.50		
4	Third	Ticagrelor tablets	Bid-winning	5.71	1.02	-82.10%***	28.00	41.08	34.14%**
			Bid-non-winning	8.45			70.00		
5	Fifth	Docetaxel injection	Bid-winning	92.67	6.45	-91.96%***	18.74	17.51	-16.04%***
			Bid-non-winning	79.22			21.03		
6	Fifth	Potassium chloride sustained-release tablets	Bid-winning	2.35	2.03	-4.18%***	7.17	7.91	10.91%***
			Bid-non-winning	1.36			7.03		

Standard errors in parentheses

** *p* < 0.05 *** *p* < 0.01

Among the 6 drugs of complete substitution, the DDDs of 3 (3/6, 50%) bid-winning brands increased significantly. Among them, the growth rate of the DDDs of ticagrelor tablets for cardiovascular system was the largest, which was 34.14% (the average value of the six months before and after policy intervention).

It was worth noting that after complete substitution, the DDDs of docetaxel injection decreased significantly while the DDDs of montelukast sodium oral granules observed no significant change. Two reasons were at play based on on-site interviews in the medical institution: For one thing, efficacy became unstable after brand substitution, therefore, doctors switched to other varieties, such as docetaxel injection; For another, the mutual substitution of policy-related drugs lead to the decrease of DDDs of bid-winning brands. Take montelukast sodium oral granules of the third round as an example, the tablets and chewable tablets of the same generic name were procured in the first and third round respectively. Within six months after policy intervention, the DDDs of these two increased by 13.22% and 24.39% respectively, which had a substitution effect on montelukast sodium oral granules.

##### Middle-intensity substitution

Middle-intensity substitution (Partial substitution) referred to that the utilization volume of bid-winning brands increased after policy intervention, gradually substituting bid-non-winning brands. In the analyzed samples, 32 drugs (32/88, 36.36%) realized partial substitution, which was the mainstream situation (Table 5 Drugs of partial substitution).

**Table 5:** Drugs of partial substitution

No.	Round	Generic Name	Brand	DDDc			DDDs		
				**Before**	**After**	**Growth Rate**	**Before**	**After**	**Growth Rate**
**27/32, 84.37% (bid-winning brands not used before NCDP and came into use after NCDP)**									
1	First	Montmorillonite powder	Bid-non-winning	2.20	2.20	0.00%***	6.34	6.30	-0.58%
			Bid-winning	/	0.83	/	/	7.60	/
2	First	Losartan potassium tablets	Bid-non-winning	5.35	4.54	-15.15%***	16.50	19.04	15.42%
			Bid-winning	/	1.02	/	/	38.17	/
3	First	Clopidogrel bisulfate tablets	Bid-non-winning	13.49	4.30	-68.10%***	14.88	17.38	16.80% ***
			Bid-winning	/	3.09	/	/	21.13	/
4	First	Irbesartan and hydrochlorothiazide tablets	Bid-non-winning	2.55	2.55	0.00%***	27.55	29.96	8.77%
			Bid-winning	/	1.02	/	/	48.55	/
5	First	Irbesartan tablets	Bid-non-winning	1.43	3.06	114.11% ***	24.83	16.74	-32.59% ***
			Bid-winning	/	0.39	/	/	16.33	/
6	First	Amlodipine besilate tablets	Bid-non-winning	6.34	5.19	-18.10% ***	15.09	12.75	-15.53% ***
			Bid-winning	/	0.09	/	/	21.84	/
7	First	Amlodipine besylate and atorvastatin calcium tablets	Bid-non-winning	15.20	14.00	-7.91%	9.11	8.90	-2.38% ***
			Bid-winning	/	1.23	/	/	11.88	/
8	Second	Bisoprolol fumarate tablets	Bid-non-winning	2.22	2.02	-8.90% ***	28.07	21.6	-23.05%
			Bid-winning	/	0.58	/	/	40.55	/
9	Third	Tamsulosin hydrochloride sustained-release capsules	Bid-non-winning	5.27	3.83	-27.29% ***	24.56	16.16	-34.20% ***
			Bid-winning	/	0.50	/	/	35.25	/
10	Third	Trimetazidine hydrochloride tablets	Bid-non-winning	4.23	4.19	-1.01% ***	22.22	15.15	-31.83%
			Bid-winning	/	0.24	/	/	19.93	/
11	Third	Moxifloxacin hydrochloride and sodium chloride injection	Bid-non-winning	216.59	32.80	-84.86% ***	5.67	15.00	164.71% **
			Bid-winning	/	32.80	/	/	9.69	/
12	Third	Ambroxol hydrochloride tablets	Bid-non-winning	2.51	2.48	-1.54% ***	4.88	4.61	-5.60% ***
			Bid-winning	/	0.34	/	/	8.82	/
13	Third	Metformin hydrochloride and glibenclamide tablets	Bid-non-winning	1.73	1.45	-16.67% ***	36.95	34.53	-6.55% **
			Bid-winning	/	0.10	/	/	59.05	/
14	Third	Valsartan capsules	Bid-non-winning	6.12	5.32	-13.15% ***	13.82	13.04	-5.62% ***
			Bid-winning	/	0.25	/	/	25.44	/
15	Third	Sodium bicarbonate tablets	Bid-non-winning	0.29	0.86	193.94% ***	58.73	66.24	12.79%
			Bid-winning	/	0.22	/	/	86.62	/
16	Third	Celecoxib capsules	Bid-non-winning	8.15	8.05	-1.26% ***	8.58	6.81	-20.72%***
			Bid-winning	/	0.59	/	/	16.46	/
17	Third	Capecitabine tablets	Bid-non-winning	118.18	165.30	39.93% ***	8.60	8.69	1.03%
			Bid-winning	/	18.40	/	/	13.50	/
18	Third	Febuxostat tablets	Bid-non-winning	19.94	14.70	-26.30% ***	10.21	7.08	-30.61%
			Bid-winning	/	1.55	/	/	15.08	/
19	Third	Domperidone tablets	Bid-non-winning	1.41	1.31	-6.87% ***	17.99	14.44	-19.76%***
			Bid-winning	/	0.50	/	/	20.68	/
20	Third	Ibuprofen sustained-release capsules	Bid-non-winning	1.30	1.26	-2.92% ***	11.33	10.86	-4.16%***
			Bid-winning	/	0.04	/	/	18.38	/
21	Third	Montelukast sodium chewable tablets	Bid-non-winning	5.08	3.82	-24.81% ***	15.54	19.33	24.39%***
			Bid-winning	/	0.16	/	/	46.01	/
22	Third	Omeprazole enteric capsules	Bid-non-winning	4.05	4.05	0.00%	16.95	15.38	-9.25%**
			Bid-winning	/	0.23	/	/	23.29	/
23	Forth	Propofol medium and long chain fat emulsion injection	Bid-non-winning	7.50	7.50	0.00%	10.00	6.60	-33.97%
			Bid-winning	47.60	46.76	-1.76% ***	3.62	4.38	20.93%***
24	Fifth	Bicalutamide tablets	Bid-non-winning	26.88	25.00	-6.99%	33.09	42.00	26.92%
			Bid-winning	/	6.89	/	/	28.00	/
25	Fifth	Budesonide suspension for inhalation	Bid-non-winning	27.79	24.32	-12.46% ***	9.49	11.81	24.37%***
			Bid-winning	/	6.38	/	/	6.25	/
26	Fifth	Miglitol tablets	Bid-non-winning	5.38	5.38	0.00%	15.02	24.00	59.80%
			Bid-winning	/	4.00	/	/	21.67	/
27	Fifth	Ipratropium bromide solution for inhalation	Bid-non-winning	8.74	8.74	0.00%	7.13	6.28	-11.96%***
			Bid-winning	/	1.94	/	/	4.84	/
**5/32, 18.75% (bid-winning brands used in small amount before NCDP** **and utilization volume increased significantly after NCDP)**									
28	First	Flurbiprofen axetil injection	Bid-non-winning	62.25	62.25	0.00%	12.84	7.31	-43.05%***
			Bid-winning	61.77	21.95	-64.46% ***	10.69	16.10	50.55%***
29	First	Dexmedetomidine hydrochloride injection	Bid-non-winning	136.55	99.97	-26.79% ***	1.58	1.38	-12.60%***
			Bid-winning	188.00	133.10	-29.20% ***	0.62	1.80	188.63%***
30	First	Rosuvastatin calcium tablets	Bid-non-winning	3.25	3.25	0.00%	22.37	19.52	-12.74%***
			Bid-winning	1.47	0.16	-89.12% ***	57.55	78.46	36.34%**
31	Fifth	Glipizide tablets	Bid-non-winning	0.90	0.90	0.00%	23.55	23.29	-1.10%
			Bid-winning	0.32	0.32	0.00%	24.00	48.00	100.00%**
32	Fifth	Saxagliptin tablets	Bid-non-winning	7.97	7.95	-0.19%	17.76	14.00	-21.15%
			Bid-winning	1.66	1.66	0.00%	30.00	33.00	10.00%**
33	Fifth	Ropivacaine hydrochloride injection	Bid-non-winning	68.55	68.55	0.00%	12.84	7.31	-43.05%***
			Bid-winning	59.46	13.76	-64.46% ***	10.69	16.10	50.55%***

Standard errors in parentheses

^**^ *p* < 0.05 *** *p* < 0.01

Among the 32 drugs of partial substitution, the bid-winning brands of 27 drugs (27/32, 84.37%) were not used before policy intervention. After policy intervention, medical institutions started using these brands. The bid-winning brands of 5 drugs (5/32, 18.75%) were seldom used before policy intervention. After policy intervention, their utilization volume significantly increased, gradually took the share of bid-non-winning brands.

#### Situation 2: no substitution

No substitution was another mainstream type of brand substitutions in the medical institution, including two situations:

##### Bid-winning brands were used both before and after policy intervention

Bid-winning brands were used both before and after policy intervention, but their DDDc and DDDs changed. This was the case for 5 (5/88, 5.68%) policy-related drugs (Table 6 Bid-winning brands were used both before and after policy intervention). Except that the DDDc of alfacalcidol tablets remained unchanged, the DDDc of other drugs all decreased significantly, the NCDP policy lowered drug prices. The DDDs of acarbose capsules and letrozole tablets decreased significantly, while other varieties observed no significant change. The possible reasons were as followed:

**Table 6:** Bid-winning brands were used both before and after policy intervention

No.	Round	Generic Name	DDDC			DDDs		
			**Before**	**After**	-76.40%***	13.48	13.14	-2.54%***
1	Second	Acarbose capsules	6.18	1.46	-62.08%***	30.25	23.18	-23.37%***
2	Third	Letrozole tablets	11.40	4.32	0.00%	15.00	26.25	75.00%
3	Fifth	Alfacalcidol tablets	2.46	2.46	-51.87%***	1.98	2.01	1.44%
4	Fifth	Iohexol injection	113.36	54.56	-21.31%***	1.28	1.21	-4.76%
5	Fifth	Misoprostol tablets	5.88	4.63	-76.40%***	13.48	13.14	-2.54%***

Standard errors in parentheses

^**^ *p* < 0.05 *** *p* < 0.01

- First, there could be mutual substitution between drugs for the same indication. Acarbose capsules and letrozole tablets had the same indication with many other policy-related drugs. For example, acarbose capsules was selected in the second round of NCDP. However, 15 more antidiabetic drugs were selected in the third to fifth round, which caused varying degrees of substitution for acarbose capsules. This caused the reduction of its DDDs.
- Second, the drug demand has weak correlation to its price. For example, iohexol injection is a contrast medium, the demand of which is directly influenced by the number of patients rather than its price. As a result, the utilization volume of iohexol injection did not significantly increase after policy intervention.

##### ① Bid-non-winning brands were used both before and after policy intervention

Bid-non-winning brands were used both before and after policy intervention, with the DDDc of bid-non-winning brands decreased. The share of bid-winning brands in the medical institution did not increase (no substitution). This was the case for 31 (31/88, 35.22%) policy-related drugs (Table 7 Bid-non-winning brands were used both before and after policy intervention).

**Table 7:** Bid-non-winning brands were used both before and after policy intervention

No.	Round	Generic Name		DDDc			DDDs		
				**Before**	**After**	**Growth rate**	**Before**	**After**	**Growth rate**
1	Fourth	Mosapride citrate tablets	Bid-non-winning	/	1.58	/	/	15.47	/
			Bid-winning	2.29	2.27	-0.91%***	13.48	11.28	-16.34%
2	Fifth	Iodixanol injection	Bid-non-winning	546.23	543.50	-0.50%***	1.15	1.17	1.74%
			Bid-winning	174.80	174.80	0.00%	2.00	1.25	-37.50%***
3	Fifth	Rivaroxaban tablets	Bid-non-winning	38.04	38.28	0.63%***	12.34	11.82	-4.21%***
			Bid-winning	0.59	0.58	-1.37%	12.22	10.80	-11.64%
4	Fifth	Ropivacaine hydrochloride for injection	Bid-non-winning	68.55	68.55	0.00%	1.52	1.17	-23.09%
			Bid-winning	59.46	13.76	-76.86% ***	1.13	1.13	-0.43%
5	Second	Hydrotalcite chewable tablets	Bid-non-winning	2.32	0.71	-69.33% ***	14.91	36.16	142.57%***
6	Second	Glimepiride tablets	Bid-non-winning	3.14	1.94	-38.18% ***	24.57	52.36	113.08%***
7	Second	Moxifloxacin hydrochloride tablets	Bid-non-winning	23.28	3.49	-84.99% ***	5.00	10.09	101.79%***
8	Second	Donepezil hydrochloride tablets	Bid-non-winning	38.23	5.73	-85.03% ***	16.33	30.01	83.74%***
9	Second	Paracetamol tablets	Bid-non-winning	0.56	0.53	-5.87% ***	22.34	21.48	-3.84%
10	Second	Tegafur gimeracil oteracil potassium capsule	Bid-non-winning	205.86	46.58	-77.37% ***	14.89	13.44	-9.75%***
11	Second	Amoxicillin capsule	Bid-non-winning	1.45	0.31	-78.42% ***	26.24	21.53	-17.97%***
12	Second	Simvastatin tablets	Bid-non-winning	2.95	2.30	-22.04% ***	20.32	14.83	-26.98%
13	Second	Candesartan cilexetil tablets	Bid-non-winning	1.43	0.45	-68.45% ***	41.72	19.79	-52.57%***
14	Third	Desloratadine tablets	Bid-non-winning	5.93	4.82	-18.81% ***	18.59	18.6	0.05%***
15	Third	Metformin hydrochloride sustained-release tablets	Bid-non-winning	2.26	2.26	0.00%	7.07	5.39	-23.76%
16	Third	Finasteride tablets	Bid-non-winning	5.57	4.10	-26.35% ***	27.58	20.02	-27.40%***
17	Fourth	Pregabalin capsule	Bid-non-winning	21.02	19.03	-9.47% ***	9.59	14.82	54.52%**
18	Fourth	Telmisartan tablets	Bid-non-winning	2.29	1.09	-52.43% ***	12.96	19.74	52.27%***
19	Fourth	Perindopril tert-butylamine tablets	Bid-non-winning	3.16	3.01	-4.88% ***	21.67	25.00	15.38%
20	Fourth	Gliclazide modified release tablets	Bid-non-winning	3.68	2.48	-32.73% ***	23.78	25.09	5.49%***
21	Fourth	Ambroxol hydrochloride injection	Bid-non-winning	12.01	0.95	-92.05% ***	16.90	17.44	3.18%***
22	Fourth	Repaglinide tablets	Bid-non-winning	2.62	2.40	-8.52%***	37.19	34.12	-8.25%
23	Fourth	Canagliflozin tablets	Bid-non-winning	3.79	3.78	-0.08%***	35.78	30.58	-14.53%
24	Fourth	Loratadine tablets	Bid-non-winning	2.91	2.91	-0.34%***	8.23	6.71	-18.57%***
25	Fourth	Ibuprofen injection	Bid-non-winning	148.80	30.87	-79.25% ***	1.60	1.22	-23.90%
26	Fourth	Esomeprazole magnesium enteric-coated tablets	Bid-non-winning	18.08	17.29	-4.33%***	11.83	8.12	-31.36%***
27	Fifth	Fluconazole and sodium chloride injection	Bid-non-winning	236.12	236.12	0.00%	9.92	19.00	91.54%
28	Fifth	Thioctic acid injection	Bid-non-winning	20.69	20.69	0.00%	44.31	52.39	18.23%***
29	Fifth	Fasudil hydrochloride injection	Bid-non-winning	91.75	91.75	0.00%	5.98	5.14	-13.97%**
30	Fifth	Levofloxacin and sodium chloride injection	Bid-non-winning	49.52	47.12	-4.85%***	6.83	5.27	-22.76%***
31	Fifth	Metoprolol tartrate tabets	Bid-non-winning	2.07	2.07	0.00%	4.76	3.47	-27.05%***

Standard errors in parentheses

^**^ *p* < 0.05 *** *p* < 0.01

Among the 31 drugs, the bid-non-winning brands of 27 drugs (27/31, 87.10%) were used both before and after policy intervention, and the DDDc of them showed a downward trend after policy intervention. Of the 27 drugs, the DDDc of 22 drugs decreased significantly, the average decrease was 39.26%. The DDDc of moxifloxacin hydrochloride tablets, donepezil hydrochloride tablets and ambroxol hydrochloride injection decreased by more than 80.00%. Moreover, the bid-non-winning brands of one drug (mosapride citrate tablets) came into use after policy intervention, causing the DDDs of the bid-winning brands decreased by 16.34%. And the DDDs of 3 drugs (iodixanol injection, rivaroxaban tablets and ropivacaine hydrochloride for injection) decreased, the DDDs reduction of their bid-winning brands was even greater than that of their bid-non-winning brands.

#### Situation 3: Alternation of varieties

##### Policy-related drugs came into use after policy intervention

Policy-related drugs came into use after policy intervention referred to drugs that were not used before policy intervention and started being used after policy intervention. This was the case for 8 (8/88, 9.10%) policy-related drugs (Table 8 Policy-related drugs used after policy intervention). The medical institution did not report the volume indicator of the bid-winning brands of azithromycin tablets and levocetirizine dihydrochloride tablets, thus keeping using the bid-non-winning brands. The bid-winning brands of other drugs were used after policy intervention.

**Table 8:** Policy-related drugs used after policy intervention

No.	Round	Generic Name	Brand	Price Reduction
1	First	Entecavir tablets	Bid-winning	79.94%***
2	First	Cefuroxime axetil tablets	Bid-winning	74.86%***
3	Second	Azithromycin tablets	Bid-non-winning	/
4	Second	Levocetirizine dihydrochloride tablets	Bid-non-winning	/
5	Second	Fudosteine tablets	Bid-winning	62.59%***
6	Third	Cefdinir capsules	Bid-winning	84.77%***
7	Third	Mecobalamin tablets	Bid-winning	97.71%***
8	Fifth	Cisatracurium besylate injection	Bid-winning	83.13%***

Retail price: the last online price of this drug in Jiangsu before the NCDP. If the drug was not sold online in Jiangsu, then the median online price of all other brands during the year before policy intervention was used

^*^ Price reduction = – (bid-winning price – retail price) /retail price *100%

Standard errors in parentheses ** *p*<0.05, *** *p*<0.01

##### Policy-related drugs no longer used after policy intervention

Six (6/88, 6.82%) policy-related drugs were no longer used by the medical institution after policy intervention (Table 9 Policy-related drugs no longer used after policy intervention). According to our analysis, three following reasons are responsible for that:

**Table 9:** Policy-related drugs no longer used after policy intervention

No.	Round	Generic Name	Brand Type	Number of Prescription Records
1	Third	Etoricoxib tablets	Bid-non-winning	185
2	Fourth	Voriconazole tablets	Bid-non-winning	23
3	Fourth	Nateglinide tablets	Bid-winning	30
4	Fifth	Tinidazole tablets	Bid-winning	23
5	Fifth	Palonosetron hydrochloride injction	Bid-non-winning and bid-winning	1457
6	Fifth	Linezolid and glucose injection	Bid-non-winning and bid-winning	31

- First, the number of medication cases of these drugs was relatively small. Voriconazole tablets, nateglinide tablets, tinidazole tablets, linezolid, and glucose injection all had less than 50 prescriptions records, which revealed that these drugs were not commonly used in the medical institution. Second, there could be mutual substitution between drugs of the same indication. For example, etoricoxib tablets was selected in the third round. Three commonly used drugs of the same indication including celecoxib capsule and paracetamol tablets were selected before or together with etoricoxib tablets, which had substitution effect on etoricoxib tablets. Third, some of these drugs became more out-patiently used rather than in-patiently used. For example, palonosetron hydrochloride injection is primarily used for preventing nausea and vomiting caused by chemotherapy. Due to the continuous improvement of the treatment level of outpatient service in China, cancer patients can apply for medical insurance reimbursement for radiotherapy, chemotherapy, and pain treatment, leading to the decrease of in-patient use of palonosetron hydrochloride injection.

### Influencing factors of drug utilization change

To probe into the influencing factors of brand substitution, three types of factors were analyzed based on literature review and field research, including policy effect, drug market condition, and previous drug utilization of the medical institution(Table 10 Influencing factors of brand substitution).

**Table 10:** Influencing factors of brand substitution

Type	Factors	Explanation	Variable Type and Assignment	References
Policy effect	Round of NCDP	Which round of the NCDP did the drug belong to	Categorical variable, the first to the fifth round were assigned as 1–5 respectively	Jiang Y(2019)^[15]^ Wang J(2022)^[16]^
Drug market condition	Price reduction of bid-winning brands	Price reduction = -(bid-winning price -retail price before policy intervention)/retail price	Continuous variable	Huang S(2019)^[17], ^ Yang X(2019)^[18]^
	Average price reduction of bid-non-winning brands	Price reduction^a^ = -(average price before policy intervention—average price after policy intervention)/average price before policy intervention	Continuous variable	
	Indication type	Indication type of the policy-related drug	Categorical variable, detailed assignment shown in Table 12	Hu S(2021)^[19], ^ Han J(2021)^[20], ^ Zhen D(2022)^[21]^
	Number of drugs of the same indication selected before	Number of policy-related drugs of the same indication selected into the NCDP before the target drug	Continuous variable	
	Number of drugs of the same indication selected after	Number of policy-related drugs of the same indication selected into the NCDP after (or together with) the target drug	Continuous variable	
Previous drug utilization of the medical institution	Whether bid-winning brands were used before policy intervention	Whether bid-winning brands were used in the medical institution before policy intervention	Binary variable	Han J(2021)^[20]^
	Whether brand-name drugs were used before policy intervention	Whether brand-name drugs were used in the medical institution before policy intervention	Binary variable	
	Number of prescriptions	Number of prescriptions from 2019 to 2021	Continuous variable	Actual situation of the medical institution

^a^Average price: the actual price in the medical institution during the statistical period

Categorical variable “indication” was assigned as below (Table 11 Influencing factors of brand utilization (indication type)):

**Table 11:** Influencing factors of brand utilization (indication type)

Assignment	Indication	Generic Name	Assignment	Indication	Generic Name
1	Alzheimer’s disease	Donepezil hydrochloride tablets	16	Prostatic hyperplasia	Finasteride tablets, Tamsulosin hydrochloride sustained-release capsules
2	Hypokalemia	Potassium chloride sustained-release tablets	17	Expectorant	Ambroxol hydrochloride injection, ambroxol hydrochloride tablets, fudosteine tablets
3	Nasids	Ibuprofen injection, Paracetamol tablets, Lbuprofen sustained-release capsules, Celecoxib capsules, Etoricoxib tablets	18	Gastrointestinal motility promoting agent	Mosapride citrate tablets, Domperidone tablets
4	Calcium metabolic disorder	Alfacalcidol tablets	19	Arthrolithiasis	Febuxstat tablets
5	Hypertension	Metoprolol tartrate tabets, candesartan cilexetil tablets, perindopril tert-butylamine tablets, telmisartan tablets, amlodipine besilate tablets, irbesartan tablets, irbesartan and hydrochlorothiazide tablets, bisoprolol fumarate tablets, losartan potassium tablets, valsartan capsules	20	Diabetes	Acarbose capsules, glimepiride tablets, gliclazide modified release tablets, canagliflozin tablets, hioctic acid injection, repaglinide tablets, metformin hydrochloride sustained-release tablets, glipizide tablets, miglitol tablets, saxagliptin tablets, metformin hydrochloride tablets, nateglinide tablets
6	Asthma	Montelukast sodium chewable tablets, budesonide suspension for inhalation, ipratropium bromide solution for inhalation, montelukast sodium oral granules, montelukast sodium tablets	21	Gastritis and peptic ulcer	Esomeprazole magnesium enteric-coated tablets, hydrotalcite chewable tablets, omeprazole enteric capsules, sodium bicarbonate tablets
7	Coronary disease	Clopidogrel bisulfate tablets, trimetazidine hydrochloride tablets, ticagrelor tablets	22	Hyperlipemia	Simvastatin tablets, atorvastatin calcium tablets, rosuvastatin calcium tablets
8	VenousThromboembolism	Rivaroxaban tablets	23	Nutrient	Propofol medium and long chain fat emulsion injection
9	Antivirals	Tenofovir disoproxil fumarate tablets, entecavir tablets	24	Contrast medium	Iohexol injection, iodixanol injection
10	Antiallergic drugs	Desloratadine tablets, loratadine tablets, levocetirizine dihydrochloride tablets	25	Sedative-hypnotic	Dexmedetomidine hydrochloride injection
11	Antibiotics	Amoxicillin capsules, fluconazole and sodium chloride injection, moxifloxacin hydrochloride tablets, levofloxacin and sodium chloride injection, moxifloxacin hydrochloride and sodium chloride injection, azithromycin tablets, cefdinir capsules, cefuroxime axetil tablets, linezolid and glucose injection, tinidazole tablets	26	Analgesia	Pregabalin capsules, flurbiprofen axetil injection
12	Antimycotic drugs	Voriconazole tablets	27	Antiemetic agents	Palonosetron hydrochloride injection
13	Anesthetic	Ropivacaine hydrochloride injection, cisatracurium besylate injection	28	Antidiarrheal drug	Montmorillonite powder
14	Peripheral neuropathy	Mecobalamin tablets	29	Interruption of ectopic pregnancy	Misoprostol tablets
15	Cerebral ischemia and cerebral vasospasm	Fasudil hydrochloride injection	30	Antineoplastic	Letrozole tablets, tegafur gimeracil oteracil potassium capsule, bicalutamide tablets, capecitabine tablets, docetaxel injection

According to results, six factors had significant influence on all situations, as shown in Table 12 (Results of parametric/non-parametric analysis) Results of parametric/non-parametric analysis:

**Table 12:** Results of parametric/non-parametric analysis

Influencing Factors		P
**Policy effect**	Round of NCDP***	0.0049
**Drug market competition**	Price reduction of bid-winning brands	0.0995
	Average price reduction of bid-non-winning brands***	0.0004
	Indication type**	0.0154
	Number of drugs of the same indication selected before	0.1912
	Number of drugs of the same indication selected after	0.9584
**Previous drug utilization of the medical institution**	Number of prescriptions***	0.0002
	Whether brand-name drugs were used before policy intervention	0.1151
	Whether bid-winning brands were used before policy intervention	0.3390

Standard errors in parentheses

^**^ *p* < 0.05 *** *p* < 0.01

#### Policy effect

In the analysis of policy effect, political factor (the order of inclusion in the NCDP) played a significant role (*P* = 0.0049). In the first and third round of NCDP, 85.71% and 69.57% policy-related drugs realized brand substitution respectively (complete substitution & partial substitution). By contrast, in the second and fourth round of NCDP, only 7.14% policy-related drugs realized brand substitution, respectively.

**Fig. 1:**
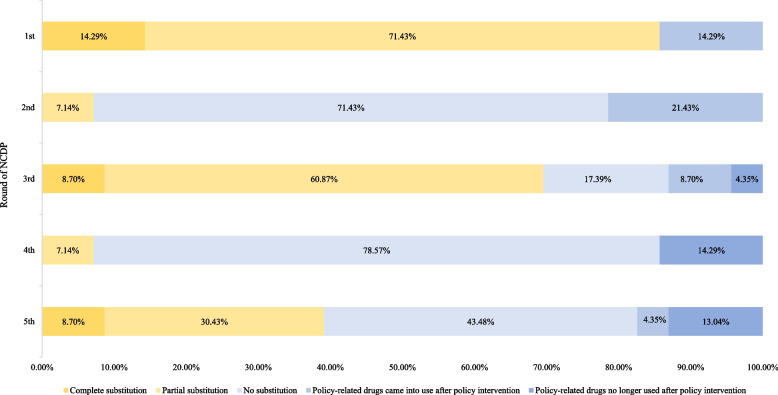
Summary of brand substitution in each round of NCDP

Our study proposed that the number of substituted drugs was positively correlated with the efforts made by government at all levels in policy promotion (Fig. 1 Summary of brand substitution in each round of NCDP).

In the first round of NCDP, of which the brand substitution rate was the highest, press conference was held to promote the pilot program before policy intervention. Policy interpretation of the NCDP implementing scheme and Q&A of rational clinical use of both bid-winning and bid-non-winning drugs were released after policy intervention. The policymakers also launched training programs on the settlement of policy-related drugs. All these efforts promoted the brand substitution in medical institutions.

For example, on January 17, 2020, Jiangsu Provincial Medical Insurance Bureau released *Notice on Issues Related to the Reasonable Clinical Use of Bid-winning and Bid-non-winning Drugs in the National Centralized Drug Procurement* to guide drug alternation. And a training meeting was held with relevant leaders from major medical institutions in Jiangsu Province, providing guidance from aspects of "priority substitution, reasonable substitution, and strengthened publicity", which helped medical institutions implement the NCDP policy.

##### **② **Drug market competition

In the analysis of drug market competition, average price reduction of bid-non-winning brands (*P* = 0.0004), indication type of bid-winning brands (*P* = 0.0154) played significant roles.

**Fig. 2:**
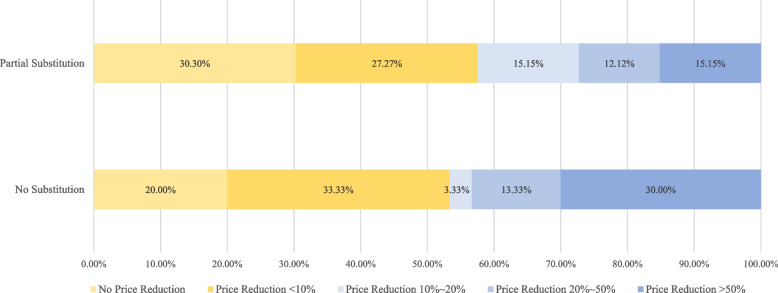
Substitution type and price reduction of bid-non-winning brands

For one thing, the tendency of brand substitution was negatively correlated with the average price reduction of bid-non-winning brands. In the case of partial substitution, the average price reduction of bid-non-winning brands was 14.29%, among which the price reduction of bid-non-winning brands of 9 drugs (27.27%, 9/33) was over 20%. By contrast, in the case of no substitution, the average price reduction of bid-non-winning brands used after policy intervention^6^ was 28.82%, among which the price reduction of bid-non-winning brands of 13 drugs (43.33%, 13/20) was over 20%. Significant difference was observed between the two situations. When bid-non-winning brands realized a relatively high price reduction, the corresponding bid-winning brands were more likely to not be substituted at all (Fig. 2 Substitution type and price reduction of bid-non-winning brands).

**Fig. 3:**
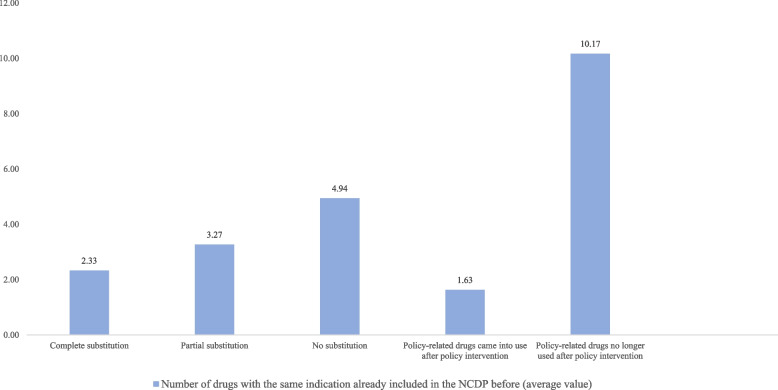
Summary of substitution type and indication type

For another, the tendency of brand substitution was negatively correlated with the number of drugs of the same indication before policy intervention. By sorting policy-related drugs of the same indication, it was observed that for the 6 policy-related drugs no longer used after policy intervention, each of them had averagely 10.17 drugs of the same indication already included in the NCDP before. Additionally, for the 35 drugs of no substitution, each of them had averagely 4.94 drugs of the same indication already included in the NCDP before. Therefore, when medical institutions had a wide selection of drugs, they tended to simplify drugs or brands (Fig. 3 Summary of substitution type and indication type).

#### Previous drug utilization of the medical institution

In the analysis of previous drug utilization of the medical institution, the number of prescriptions was positively correlated with the tendency of brand substitution (*P* = 0.0002).

Drugs with large number of prescriptions were more likely to realize partial substitution. Because relatively larger utilization volume and higher frequency of use allowed new brands to enter the market or change the original market share. In our study, 33 drugs (33/88, 37.50%) realized partial substitution, their average number of prescriptions was 3,034.

However, drugs with relatively moderate utilization volume and frequency of use were more likely to realize complete or no substitution. To drugs of complete and no substitution, the average number of prescriptions was 1,608 and 1,518 respectively, about 50% of partial substitution. For these drugs, medical institutions preferred to choose a specific brand, resulting in complete or no substitution.

Apart from the number of prescriptions, our study found in the exploratory interview that the tendency of brand substitution was negatively correlated with the preference for brand-name drugs, in other words, the proportion of brand-name prescriptions was relatively large after policy intervention. Drugs with weaker preference for brand-name drugs were more likely to realize brand substitution. In the 6 types of brand substitution, 32 drugs (32/88, 36.36%) had used brand-name drugs before policy intervention, 2 of which (2/32, 6.25%) realized complete substitution. The proportion of brand-name drug prescriptions of these 2 drugs turned from 78.26% to 0% after policy intervention, which showed extremely weak preference for brand-name drugs.

Fourteen drugs (14/32,43.75%) realized partial substitution. The proportion of brand-name drug prescriptions of these 14 drugs dramatically decreased after policy intervention, turning from 78.15% to 31.17%, which showed relatively weak preference for brand-name drugs.

**Fig. 4:**
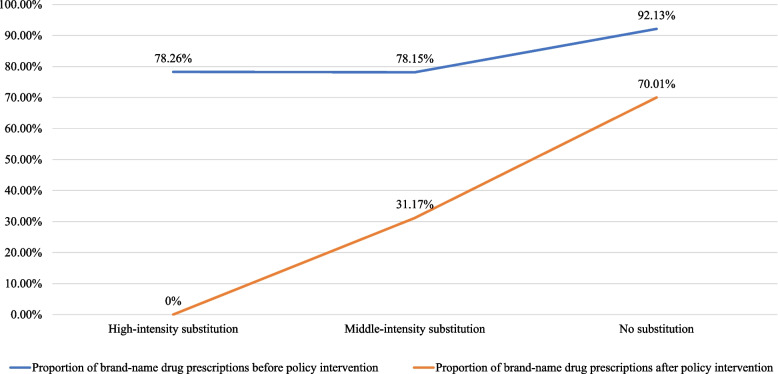
Relation between substitution intensity and preference for brand-name drugs

Fifteen drugs (15/32, 46.87%) realized no substitution (bid-non-winning brands were used both before and after policy intervention).^7^ The proportion of brand-name drug prescriptions of these 15 drugs turned from 92.13% to 70.01% after policy intervention. Among them, esomeprazole magnesium enteric-coated tablets, finasteride tablets, and other 8 drugs still only used brand-name drugs after policy intervention, showing strong preference for brand-name drugs. To conclude, in actual medication, some drugs had strong preference for brand-name drugs, directly weakening the substitution of bid-winning brands (Fig. 4 Relation between substitution intensity and preference for brand-name drugs).

## Discussion

### The NCDP policy influenced the medication selection of medical institutions

The NCDP policy altered the medication behavior of medical institutions through the substitution of bid-winning brands for bid-non-winning brands. 43.18% of the 88 policy-related drugs realized brand substitution after policy intervention (6.82% of complete substitution and 36.36% of partial substitution). The NCDP policy effectively promoted brand substitution in medical institutions.

Meanwhile, 39.77% policy-related drugs realized no substitution. Our study proposed that it was due to the following reasons:

First, the price of bid-non-winning brands dropped significantly. The average price reduction was 28.82%, and for some drugs it exceeded 60%. For example, prices of hydrotalcite chewable tablets and donepezil hydrochloride tablets dropped by 69.33% and 89.03% respectively. As a result, medical institutions maintained the original brand selection. Second, field research found that the target medical institution did not report the quantity demand of some policy-related drugs, thus being free from the assessment pressure of using bid-winning brands. For example, among drugs of no substitution, 4 drugs (thioctic acid injection, fluconazole and sodium chloride injection, desloratadine tablets, esomeprazole magnesium enteric-coated tablets) did not have the indicator for the utilization volume of bid-winning brands. Therefore, the medical institution did not change its brand selection into bid-winning brands.

### Multiple factors influenced the substitution of bid-winning brands for bid-non-winning brands

According to analysis, three kinds of factors had significant impact on the substitution of bid-winning brands for bid-non-winning brands: (1) policy effect, including round of the NCDP, (2) drug market competition, including price reduction of bid-non-winning brands and indication type, (3) previous drug utilization of the medical institution, including number of prescriptions and the preference for brand-name drugs.

From the perspective of policy effect, the efforts made by government at all levels in policy implementation could influence brand substitution, which was obvious in the first and third round. However, because the NCDP policy is directed by the NHSA while medical institutions are appraised by the NHC, we suggest that the coordination between the NHSA and the NHC should be strengthened. Enough time should be secured for policy implementation, and the NCDP policy appraisal system implemented by NHSA should be coordinated with the medical institutions appraisal system implemented by NHC. That is, the two appraisal systems are used together to evaluate the effect of the NCDP policy, urging medical institutions to give priority to using policy-related drugs and bid-winning brands, thus lightening the medication burden of patients.

From the perspective of drug market competition, the greater the price reduction of bid-non-winning brands, the more the drugs of the same indication selected before, the more likely that medical institutions tended to use previous brands.

For one thing, policy-related drugs whose bid-non-winning brands’ prices reduced significantly were less likely to realize brand substitution. Thus, it can be seen, the NCDP policy featured strong positive externality. It promoted the gradient price reduction of bid-non-winning brands, in the long term, the NCDP policy could help reduce sales expenses and purify market environment.

For another, the indications of policy-related drugs showed high degree of repetition. Therefore, our study suggested that the selection of policy-related drugs should be guided by clinical need and give priority to diseases with relatively fewer policy-related drugs, such as ophthalmology and cerebrovascular diseases. In this way, the scope of diseases covered by policy-related drugs could be expanded, thus benefiting wider patient groups.

From the perspective of previous drug utilization of the medical institution, brand substitution was more obvious in drugs with less brand-name preference and larger utilization volume.

Brand-name preference was an important influencing factor of brand substitution. For policy-related drugs of no substitution, brand-name drug prescriptions accounted for 92.31% before policy intervention and 70.01% after policy intervention. By contrast, for policy-related drugs of partial substitution, brand-name drug prescriptions accounted for 78.15% before policy intervention and dropped to 31.17% after policy intervention. Therefore, although the NCDP policy improved the substitution rate of generic drugs, certain policy-related drugs with strong brand-name preference still failed to realize the substitution of generic drugs.

### Advantages and limitations

Our study had the following limitations due to its design. First, our study was based on one county-level medical institution. Considering the differences of economic level among regions, the differences of drug utilization habits among medical departments and drug market competition, our study results could not represent the overall situation of county-level medical institutions in China. Second, our study only used inpatient data, excluding outpatient information, which posed limitations as policy-related drugs were used in both settings.

Despite the abovementioned limitations, our study built up connections between multiple rounds of NCDP and the drug utilization of medical institutions, evaluated whether the utilization volume of policy-related drugs and different brands of the target medical institution changed after the implementation of multiple rounds of NCDP.

Furthermore, our study was based on a county-level medical institution. County-level medical institutions have the widest distribution in China, thus reflecting the influence of the NCDP policy on brand substitution and the policy effect on an universal basis. Therefore, our study has reference value for the quantitative study of the NCDP policy and further policy improvement.

## Conclusion

The NCDP policy promoted the substitution of bid-winning brands and increased their utilization volume, lowered overall drug prices, benefited pharmaceutical companies and patients, realized the initial intention of exchanging quantity for low prices and lightening patient burden. However, the NCDP policy remained to be further implemented in county-level medical institutions. Policy enforcement, drug market competition and drug utilization of medical institutions would affect the implementation of the NCDP policy.

## Data Availability

The datasets analyzed during the current study are not publicly available because they were obtained from Hospital Information System, but they are available from the corresponding author on reasonable request. And the raw data did not require any administrative permission.

## Abbreviations

AUD: Antibiotics Use Density
CNY: Chinese Yuan
DID: Differences-in-differences model
DDDs: Defined Daily Doses
DDDc: Defined Daily Dose Cost
ITS: Interrupted Time Series
NCDP: National Centralized Drug Procurement
NHSA: National Healthcare Security Administration
NHC: National Health Commission

## Footnotes

1 It refers to a management platform established by the National Healthcare Security Administration (NHSA)(https://pub.smpaa.cn/login?rn=1). This platform is responsible for managing the entire process of procurement, including tasks such as the submission of procurement requirements from healthcare institutions, prequalification of enterprises, the bidding process and so on.

2 The price of the bid-non-winning drug is the average DDDc of all non-winning brands in the target medical institutions during research cycle.

3 West China Hospital of Sichuan University: remaining at its post to fight Covid 19, Anesthesia Surgery Center is in action[EB\OL]http://www.wchscu.cn/detail/65199.html

4 Published by Taizhou High-tech District: notice of suspending the service of medical institutions including outpatient departments and clinics[EB\OL] https://mp.weixin.qq.com/s?__biz=MzI2NzA5NDk2Ng==&mid=2657311916&idx=4&sn=af74bcce6a9b23dec019d7bf7689b977&chksm=f113e275c6646b63a308fc2762a12820ec6c9c9cccac80d6df878d3f5b235c2238ce4a543dc2&scene=27

5 commonly-used policy-related drugs: policy-relates drugs whose number of prescription in the research cycle is greater than 20.

6 Because in the situation of “bid-winning brands were used both before and after policy intervention”, price data of bid-non-winning brands were unavailable. To ensure that the result was reliable, only the price reduction of bid-non-winning brands in the situation of “bid-non-winning brands were used both before and after policy intervention” was evaluated.

7 Because during the study period, the 5 drugs of “policy-related drugs were used both before and after policy intervention” used bid-winning generic drugs only. Therefore, to ensure that the result was reliable, only the situation of “bid-non-winning brands were used both before and after policy intervention” of no substitution was used to evaluate the preference for brand-name drugs.
